# Cross sectional analysis of Chicago vs suburban Cook County suicide deaths among 10–24-year-olds in the Illinois violent death reporting system

**DOI:** 10.1186/s40621-018-0142-2

**Published:** 2018-04-10

**Authors:** Ernika G. Quimby, Suzanne G. McLone, Maryann Mason, Karen Sheehan

**Affiliations:** 1 Division of Emergency Medicine, The Children’s Hospital of Philadelphia, Philadelphia, PA USA; 2 Injury Prevention and Research Center, Ann & Robert H. Lurie Children’s Hospital of Chicago, Chicago, IL USA

**Keywords:** Suicide, Young adults, Violent death

## Abstract

**Background:**

In 2014, suicide was the second leading cause of death among 10- to 24-year-olds in the US. Studies note disparities in youth suicide based on sex, race/ethnicity, and urban vs rural settings. This study investigates demographics, mental health indicators, and other circumstances surrounding youth/young adult deaths by suicide, comparing Chicago and suburban Cook County from 2005 to 2010.

**Methods:**

Using the Illinois Violent Death Reporting System (IVDRS), we employed a cross-sectional design to provide descriptive analysis of decedents in three age groups (10–14, 15–19, and 20–24 years) in two geographic areas: urban (city of Chicago) and suburban (suburban Cook County) between January 1, 2005 and December 31, 2010. We used chi-square testing to test for significant differences in each age group by demographics, mental health indicators, and suicide markers in each area.

**Results:**

Between 2005 and 2010, the IVDRS reported 299 deaths by suicide among 10–24-year-olds, 52% in Chicago, and 48% in suburban Cook County. Of these deaths, 5.7%, 33.4% and 60.9% were ages 10–14, 15–19, and 20–24 years, respectively. Non-Hispanic (NH) whites comprised 50.7% of the totals, NH Blacks 26.5%, Hispanics 16.8%, and Asians 5.7%. In Chicago, males were 84% of suicides and 62.7% in suburban Cook County among 15–19-year-olds (*p* < 0.05). White race was significantly different in 10–14-year-olds: 0% in Chicago, 54% in suburban Cook County (*p* < 0.05). Racial and ethnic differences in suicides among 15–19-year-olds in Chicago vs suburban Cook County were: NH White 22.4% vs 74.5% (*p* < 0.001), NH Black 46.9% vs 13.7% (*p* < 0.05), Hispanic 24.5% vs 7.8% (*p* < 0.05). There were also differences for 20–24-year-olds with NH White 43% vs 65.4% and NH Black 32% vs 13.6% (*p* < 0.05 for both). For mechanism of death, in 15–19-year-olds, there were differences between city and suburban in firearm deaths (42.9% vs 20%, *p* < 0.05) and in poisoning (0 vs 14%, *p* < 0.05).

**Conclusions:**

Our analyses detected significant location-related differences in the characteristics of decedents within the Chicago region indicating that local data are needed to inform suicide prevention efforts so that those at most risk can be prioritized for services. IVDRS is a potent tool in identifying these variations.

## Background

In 2014, suicide was the second leading cause of death among 10 to 24-year-olds in the United States (Centers for Disease Control and Prevention, 2018). In 2004, suicide was the third leading cause of death in this age group (Lubell et al., 2007). More specifically, WISQARS data show suicide rates significantly increased in the United States between 2005 and 2010 among 10–14-year-olds, 15–19-year-olds and 20–24-year-olds, by 52%, 28%, and 22%, respectively. Suicide has become an increasingly high cause of death in Illinois metropolitan areas (which include Cook County) among 10–14-year-olds, rising from the 8th leading cause of death in 2005, to fifth in 2010, and finally to the number one leading cause of death in 2015 for this age group. Among all youth aged 10–24 years in the Illinois metropolitan area, the suicide rate per 100,000 increased from 4.2% in 2005 to 5.6% in 2010 to 7.9% in 2015. Significant increases have occurred during this time period for Non-Hispanic White youths in all three of these age groups, and in Non-Hispanic Black youths in 15–19-year-olds. These data indicate that suicide is a serious and persistent health risk for youth and young adults in the United States, and specifically in metropolitan areas in Illinois.

Studies have noted disparities in youth suicide based on sex, race/ethnicity and circumstances surrounding death, including location. These disparities point to the need for strategies targeting factors contributing to suicide deaths in youth/young adults, especially among disproportionately affected groups (Karch et al., 2013; McLone et al., 2016; Bridge et al., 2015; Fontanella et al., 2015). For example, national data sets have found most decedents are male (Karch et al., 2013). Age related differences have been documented, including differences in method of suicide death and association of a preceding crisis (McLone et al., 2016). With regard to racial disparities, between 1993 and 2012, though the overall suicide death rate among children aged 5–11 years was stable, suicide significantly increased for Black children and significantly decreased for White children (Bridge et al., 2015). There is also evidence that the setting in which youth live may be associated with suicidality trends. For example, urban-rural disparities, have also been identified, with studies finding higher youth and young adult suicide rates in rural communities (Fontanella et al., 2015). This study investigates demographics, mental health indicators, and other circumstances surrounding youth/young adult deaths by suicide, comparing Chicago and suburban Cook County from 2005 to 2010 to begin to better identify trends in recent years.

## Methods

This study was cross-sectional, using the Illinois Violent Death Reporting System (IVDRS), a surveillance system containing information on violent deaths including suicide, homicide, legal intervention, and unintentional firearm deaths in Illinois. IVDRS collects data from death certificates, coroner/medical examiner, law enforcement, and toxicology reports to identify the circumstances surrounding each death (IVDRS 2015). The IVDRS is based on the National Violent Death Reporting System (NVDRS). The NVDRS is a state-based surveillance system developed by the Centers for Disease Control and Prevention, National Center for Injury Prevention and Control (CDC Injury Center). NVDRS collects data on violent deaths, including decedent demographics, mechanism of injury, method of injury, location of residence, location of injury, location of death, autopsy and toxicology results, and circumstances surrounding the death. The primary data sources are death certificates, coroner/medical examiner reports, law enforcement reports and crime lab reports; data are linked by decedent and entered into a web-based data repository.

The IVDRS collects violent data from a sample of urban, suburban and rural counties in Illinois. From 2005 to 2014, the IVDRS operated as a shadow system as it used CDC Injury Center software and protocols to collect data, but no funding or official support was received from the CDC Injury Center. IVDRS data collected prior to January 1, 2015 are not in the CDC’s Web-based Injury Statistics Query and Reporting System (WISQARS), which is an interactive, online database that provides fatal and nonfatal injury, violent death, and cost of injury data from a variety of trusted sources. From 2005 to 2010 the IVDRS only collected data from five Illinois counties, including Cook County, Illinois, which contains the City of Chicago. For the years 2011–2014 only some basic data elements were collected; therefore, data from these years were insufficient to be included in this present study.

The data used for this analysis included all data contained in IVDRS for calendar years 2005–2010, which represents all data collected on the decedents during this time period as of June 2014. Decedent cases were selected according to the following criteria: manner of death was suicide, decedents were between the ages of 10 and 24 years, and both the injury and subsequent death occurred in Cook County.

All decedent cases were separated into the following age groups, in years: 10–14, 15–19, and 20–24. We described demographics, method of suicide, and the presence of 13 separate circumstances among these age groups: Suicide occurred in own residence, positive alcohol test, problems with current or former intimate partner, other relationship problem(s) with family member/friend/associate, problems at school, crisis within two weeks of suicide, current depressed mood (not a diagnosis), current mental health diagnosis, currently in treatment for a mental health issue, ever treated for a mental health issue (diagnosed), history of prior suicide attempt, disclosed intent to commit suicide, and presence of a suicide note. All variables were described using percentages. Differences between urban (City of Chicago) and suburban (remainder of Cook County) were assessed using Pearson’s χ^2^.

Data were collected in software developed by the CDC for NVDRS; all analyses were performed using SPSS 22.0 (IBM, Chicago, IL).

The study was deemed exempt from Institutional Review Board review.

## Results

Between 2005 and 2010, IVDRS reported 299 deaths by suicide among 10–24-year-olds, 52% of whom were in Chicago, and 48% in suburban Cook County. Non-Hispanic whites comprised 51% of the totals, Non-Hispanic Blacks 26%, Hispanics 17%, and Asians 6%. Hanging/strangulation was the most common mechanism of death at 44%, followed by firearm (29%) and poisoning (12%). Amongst the 299 suicide deaths, 6%, 33% and 61% were ages 10–14, 15–19, and 20–24 years, respectively. We compared Chicago and suburban Cook County within each of the three age groups.

Demographic characteristics included sex and race/ethnicity (Table 1). The majority of suicide deaths in both the Chicago and suburban groups were males across all age groups. However, male/female percentages were significantly different between Chicago and suburban Cook County. In Chicago, males made up 84% of suicides, and 63% in suburban Cook County, among 15–19-year-olds (*p* < 0.05).

**Table 1:** Participant characteristics by location and age group

	10 to 14			15 to 19			20 to 24		
	*n* = 17			*n* = 100			*n* = 182		
	Chicago	Suburban Cook		Chicago	Suburban Cook		Chicago	Suburban Cook	
Variable	%	%		%	%				
Sex									
Male	66.7	81.8		83.7	62.7	*p < 0.05*	80.2	81.5	
Female	33.3	18.2		16.3	37.3		19.8	18.5	
Race/Ethnicity									
NH White	0.0	54.5	*p < 0.05*	22.4	74.5	*p < 0.05*	43.0	65.4	*p < 0.05*
NH Black	50.0	27.3		46.9	13.7	*p < 0.05*	32.0	13.6	*p < 0.05*
Hispanic	50.0	18.2		24.5	7.8	*p < 0.05*	18.0	13.6	
Means/Method									
Firearm	16.7	9.1		42.9	20.0	*p < 0.05*	33.0	25.0	*p < 0.05*
Hanging/Strangulation	83.3	72.7		40.8	52.0		33.0	48.8	
Poison	0.0	9.1		0.0	14.0	*p < 0.05*	13.0	18.8	

There was significant variation in the proportion of decedents’ race/ethnicity between the urban (Chicago) and suburban (suburban Cook County) locations, in all three age groups. The proportion of decedents who were non-Hispanic White was significantly different between the two locations among 10–14-year-olds, with 0% in Chicago and 54% in suburban Cook County (*p* < 0.05). Among 15–19-year-olds, the proportion of decedents by race/ethnicity for non-Hispanic (NH) Whites, NH Blacks, and Hispanics were each significantly different between Chicago and suburban Cook County (NH White 22% vs 74%, *p* < 0.05; NH Black 47% vs 14%, *p* < 0.05; Hispanic 24% vs 8%, *p* < 0.05). Finally, statistically significant differences were found for the proportion of NH White (43% vs 65% for Chicago and suburban Cook County, respectively) and NH Black (32% vs 14%) (*p* < 0.05 for both) decedents among 20–24-year-olds.

Hanging/strangulation, firearms, and poisoning, in that order, were the most common means/methods of suicide deaths in all three of the age groups, in both Chicago and suburban Cook County (Table 1). While no statistically significant differences between locations were found for method of death in ages 10–14 years, there were significant differences in the means of death between locations in the other two age groups. In 15–19-year-olds, there were statistically significant differences between Chicago and suburban Cook County in firearm deaths (43% vs 20%, *p* < 0.05) and in poisoning deaths (0% vs 14%, *p* < 0.05). Finally, in 20–24-year-olds, only firearm deaths were significantly different between Chicago and suburban Cook county (33% vs 25%, *p* < 0.05).

Results of the circumstances surrounding each death are presented in Table 2. There were no statistically significant differences between the two locations across any age groups with regard to whether the incident occurred at the decedent’s residence (the majority did in all groups, though the percentage decreased in both locations among the older ages) or whether the decedent tested positive for alcohol (the majority did not in all groups, though the percentage increased in both locations among the older ages). Several other factors were investigated, including whether the decedent had problems with a current or former intimate partner, other relationship problem(s) with a family member/friend/associate, problems at school, or if he or she had experienced a crisis within two weeks of suicide, but none were significantly different between Chicago and suburban Cook County in any of the age groups.

**Table 2:** Circumstances surrounding suicide and factors contributing to suicide by location and age group

	10 to 14		15 to 19		20 to 24	
	*n* = 17		*n* = 100		*n* = 182	
	Chicago	Suburban Cook	Chicago	Suburban Cook	Chicago	Suburban Cook
Variable	%	%	%	%	%	%
Circumstances						
Occurred at decedent’s residence	83.3	81.8	65.3	70.6	58.4	66.7
Positive for alcohol	0.0	0.0	16.7	30.6	42.1	46.4
Factors which appeared to have contributed to suicide						
Problems with current/former intimate partner	0.0	9.1	22.4	15.7	26.7	16.0
Other relationship problem(s)	33.3	36.4	18.4	17.6	6.9	7.4
Problems at school	16.7	27.3	4.1	2.0	1.0	4.9
Victim experienced crisis within 2 weeks of suicide	33.3	27.3	14.3	13.7	21.8	21.0

Multiple mental health indicators, including diagnosed mental health problems and noted, but not necessarily diagnosed, depressed mood, were analyzed (Table 3). In 20–24-year-olds, in Chicago, 29% had a current diagnosed mental health problem compared with 47% in suburban Cook County (*p* < 0.05). In 10–14-year-olds, two variables were significant: currently or history of ever being treated for a mental health problem (both variables were 0% in Chicago vs 36% in suburban Cook County, *p* < 0.05). Current depressed mood did not show any significant difference. We also analyzed specific suicide concerns. The decedent disclosing an intent to commit suicide to another person was significantly different between Chicago and suburban Cook County only among 10–14-year-olds (0% vs 36%, *p* < 0.05). The decedent leaving a suicide note was significantly different between Chicago and suburban Cook County only among 15–19-year-olds (22% vs 45%, *p* < 0.05). There was no significant difference between the two locations found for decedents having a history of prior suicide attempts. The majority of decedents, across both locations and ages, did not have a recorded mental health or suicide history.

**Table 3:** Mental health and suicide indicators by location and age group

	10 to 14			15 to 19			20 to 24		
	*n* = 17			*n* = 100			*n* = 182		
	Chicago	Suburban Cook		Chicago	Suburban Cook		Chicago	Suburban Cook	
Variable	%	%		%	%		%	%	
Mental Health	0.0	27.3		38.8	21.6		20.8	24.7	
Current depressed mood (not a diagnosis)									
Current mental health diagnosis	16.7	45.5		36.7	35.3		28.7	46.9	*p < 0.05*
Currently in treatment for mental health diagnosis	0.0	36.4	*p < 0.05*	24.5	29.4		13.9	21.0	
History of ever being treated for mental health issue (diagnosed)	0.0	36.4	*p < 0.05*	28.6	33.3		18.8	29.6	
Suicide									
History of prior suicide attempts	16.7	18.2		16.3	13.7		18.8	14.8	
Disclosed intent to commit suicide	0.0	36.4	*p < 0.05*	20.4	25.5		11.9	16.0	
Suicide note	0.0	18.2		22.4	45.1	*p < 0.05*	22.8	24.7	

## Discussion

Overall, we found several significant differences by race/ethnicity, sex, and mental health indicators between the urban and suburban location for each youth age group. These findings have implications for how to best tailor suicide prevention services to distinct populations taking into account race/ethnicity, age and location by highlighting different features that could potentially be used to identify and target high risk youth populations for services, as the need for further research and identification of trends continues (Gould et al., 2003). Additionally, prior studies have indicated that though both parents and youth recognize that suicide is an important problem, they often think that it is a problem in other communities, not their own (Schwartz et al., 2010). Identification of common features more specific to communities could thus help personalize information presented to parents and youth themselves, not just those providing services.

In our findings, across age groups, there were significantly more White decedents in the suburban setting. Among older decedents aged 15–19-years old, there were significantly more Blacks and Hispanics in the city setting, and additionally more Black decedents in the city among 20–24-year-olds. Multiple theories have been described for this, including increased stress on youth of color relating to racism, and a lack of access to resources at the individual and family levels; a “double risk of death” due to high rates of homicide and suicide amongst Black men also emphasizes the need for more identification and intervention of risk (Baker, 1990).

Method of death warrants further discussion from a preventive stance. In our study, firearm deaths were more common in the city than suburban settings, with statistical difference found in older youth above 15 years old. More research is needed to better elucidate related factors, such as access to guns in the different settings, as well as storage of guns in homes that do have them. Studies have shown a significantly increased risk of suicide when guns are accessible, even when controlling for confounders (Miller et al., 2007; Birckmayer & Hemenway, 2001). The increased risk of suicide death with gun access may be even higher among youth than older adults; for example, one study showed a statistically significant increase for 15–24-year olds after controlling for such factors as education, poverty, and unemployment, but the same relationship was not seen among 25–44 year olds (Birckmayer & Hemenway, 2001). In terms of access, another study highlighted that the majority of adolescent suicide deaths are committed using a family member’s gun (Johnson et al., 2010). Prior research has indicated that among households that have a gun, the risk of both intentional and non-intentional injuries may be reduced with safe-storage methods, with the gun locked and unloaded with ammunition stored locked in a different location (Grossman et al., 2005).

Although no differences were noted between the groups’ circumstances and surrounding factors, prior studies indicate an association between youth suicide and factors such as comorbid alcohol abuse or family discord (Cash & Bridge, 2009). Several more specific mental health indicators did reveal differences. Suburban youth in the youngest age group, 10–14-year-olds, were more likely to be currently or previously receiving treatment for a mental health issue than urban children in the same age group. This may be related to the lack of mental health services for youth, especially in locations with limited resources, and highlights the need for providers besides specialized mental health practitioners, such as pediatricians to assess suicide risk in youth with whom they interact (Shain, 2007).

Limitations of this study include the small sample size that was present in the database. Additionally, subjects were grouped into two large categories “Chicago” and “Suburban Cook County”, though both areas have significant diversity within each subgroup. The age of the data is another limitation; due to the constraints of the data available through the IVDRS, only data through 2010 was available for use. The mental health indicator, “Current depressed mood” is not a diagnosis and is dependent on next of kin reporting. This may lead to under reporting of depressed mood. The data were all retrospectively obtained through the IVDRS database, for deaths determined to be suicides. Data abstractors are limited to the data included in the reports they receive. Reports might not fully reflect all information known about an incident. Although extensive coding training is conducted and help desk support is available daily, variations in coding might occur depending on the abstractor’s level of experience. For this reason, IVDRS regularly conducts blinded reabstraction of cases to test consistency and identify training needs. Protective factor data (i.e., characteristics or circumstances that reduce the risk for violent death) are not collected by NVDRS as a result of the nature of death certificate, CME record, and police reports, which typically contain only circumstances associated with risk factors. Finally, medical and mental health information (e.g., type of condition and whether the decedent was currently receiving treatment) are not often captured directly from medical records but from coroner/medical examiner reports and the decedent’s family members and friends. Therefore, the completeness and accuracy of this information are limited by the knowledge of the informant (Karch et al., 2009; Lyons et al., 2016).

## Conclusion

Our analyses detected significant differences by location among youth suicide decedents within the Chicago region. These findings indicate that local data, such as the IVDRS, may help to inform suicide prevention efforts by identifying high risk populations of youth within specific regions. Future research is warranted to further explore reasons for these differences in demographic and situational circumstances for youth suicide to better inform local prevention efforts. Additionally, specific to the IVDRS, research should identify additional qualitative findings to further elucidate patterns of suicide in urban and suburban Chicago settings.

## Abbreviations

IVDRS: Illinois Violent Death Reporting System
NVDRS: National Violent Death Reporting System
